# A popliteal giant synovial osteochondroma mimicking a parosteal osteosarcoma

**DOI:** 10.1186/1477-7819-11-241

**Published:** 2013-09-25

**Authors:** Andreas Toepfer, Florian Pohlig, Heinrich Mühlhofer, Florian Lenze, Rüdiger von Eisenhart-Rothe, Ulrich Lenze

**Affiliations:** 1 Klinik für Orthopädie und Sportorthopädie, Klinikum rechts der Isar der Technischen Universität München, Ismaningerstr. 22, 81675 Munich, Germany

**Keywords:** Giant synovial osteochondroma, Chondromatosis, Bone tumor, Synovial metaplasia, Parosteal osteosarcoma

## Abstract

Both giant synovial osteochondroma and parosteal osteosarcoma are rare musculo-skeletal tumors, often localized in the vicinity of the knee. Misdiagnosis of a malignant bone tumor can entail fatal consequences. Etiology of giant synovial osteochondroma is widely unsolved but is believed to originate from synovial chondromatosis, a mostly benign metaplasia of the synovial membrane. Parosteal osteosarcoma is a low-grade surface osteosarcoma with a propensity of local recurrence and the potential of distant metastasis and therefore requiring a different therapeutical approach. We report the case of a popliteal giant osteochondroma mimicking a parosteal osteosarcoma. Relevant facts of this rare entity regarding pathogenesis, treatment, and differential diagnoses will be discussed.

## Background

For benign lesions of the bone, no reliable data regarding incidence rates are available. A large amount of benign bone tumors remains undiscovered due to an asymptomatic course. Many diagnoses of benign bone lesions are incidental findings. Malignant primary bone tumors are even less frequent, accounting for only 0.2% of all neoplasms [1]. Classic, high-grade osteosarcoma is the most common primary malignant tumor of bone (35% of all bone sarcomas), parosteal osteosarcoma is far less frequent though, amounting to only 5% of all osteosarcomas. The second most common localization for parosteal osteosarcoma is the proximal tibia, after the distal metaphysis of the posterior femur. Most patients are young adults. A limitation of flexion of the affected knee due to a painless swelling may be the initial symptom. Complete excision is mandatory to avoid recurrence and progression to high-grade osteosarcoma [2].

Giant synovial osteochondroma is a very rare form of an osteochondromatous tumor and not to be confused with classic osteochondroma (osteo-cartilaginous exostosis), the most common bone tumor. Pathogenesis of this lesion is related to a synovial metaplasia in most cases. Descriptions are limited to case studies although its vicinity to large joints and tendons implies a high rate of mechanical impairment. Predilection site is the intra-articular, infrapatellar region of the knee. Besides an intra-articular form, which is often named giant synovial osteochondroma, an equally rare extra-synovial form does exist. It preferentially appears in the tendonsheath-rich areas of hand and foot or the knee and is also referred to as extraskeletal chondroma.

Differentiation between parosteal osteosarcoma and giant synovial osteochondroma proves to be difficult as localization, clinical course, and imaging can show remarkable similarities.

## Case presentation

A 39-year-old Caucasian man presented with a painful swelling of the right popliteal space, which he first noticed 6 months ago. Patient’s history indicated no injury to the leg.

Physical examination revealed an approximately 6×4 cm hard and slightly tender mass palpable at the medial aspect of the popliteal space adjacent to the pes anserine. The mass was firm on palpation and immobile to the deep tissue. There were no inflammatory signs whatsoever. The functional testing of the knee joint showed a limited range of motion with a moderate flexion deficit of 120-0-0 °F/E. There was no pain during rotation and compression and the ligamentous structures showed sufficient stability. No signs of an intra-articular effusion were observed. Muscle strength and neurovascular examination of the leg were normal. No further, similar masses were noted during the general examination as well as no lymphadenopathy. The remainder of the physical examination and routine blood investigations, including uric acid levels, alkaline phosphatase, and inflammatory parameters (C-reactive protein, white blood cell count) were unremarkable.

**Figure 1:**
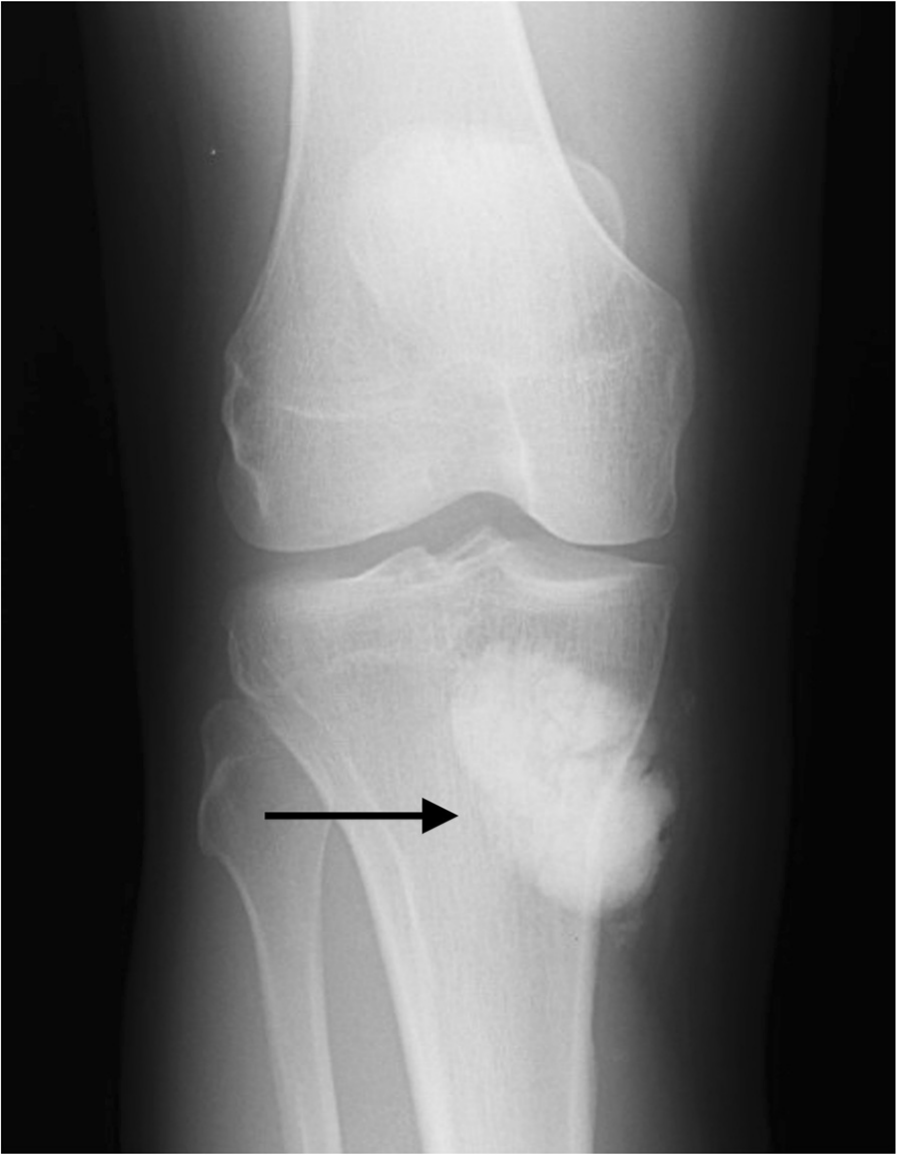
Anterior-posterior plain radiograph of the lesion.

**Figure 2:**
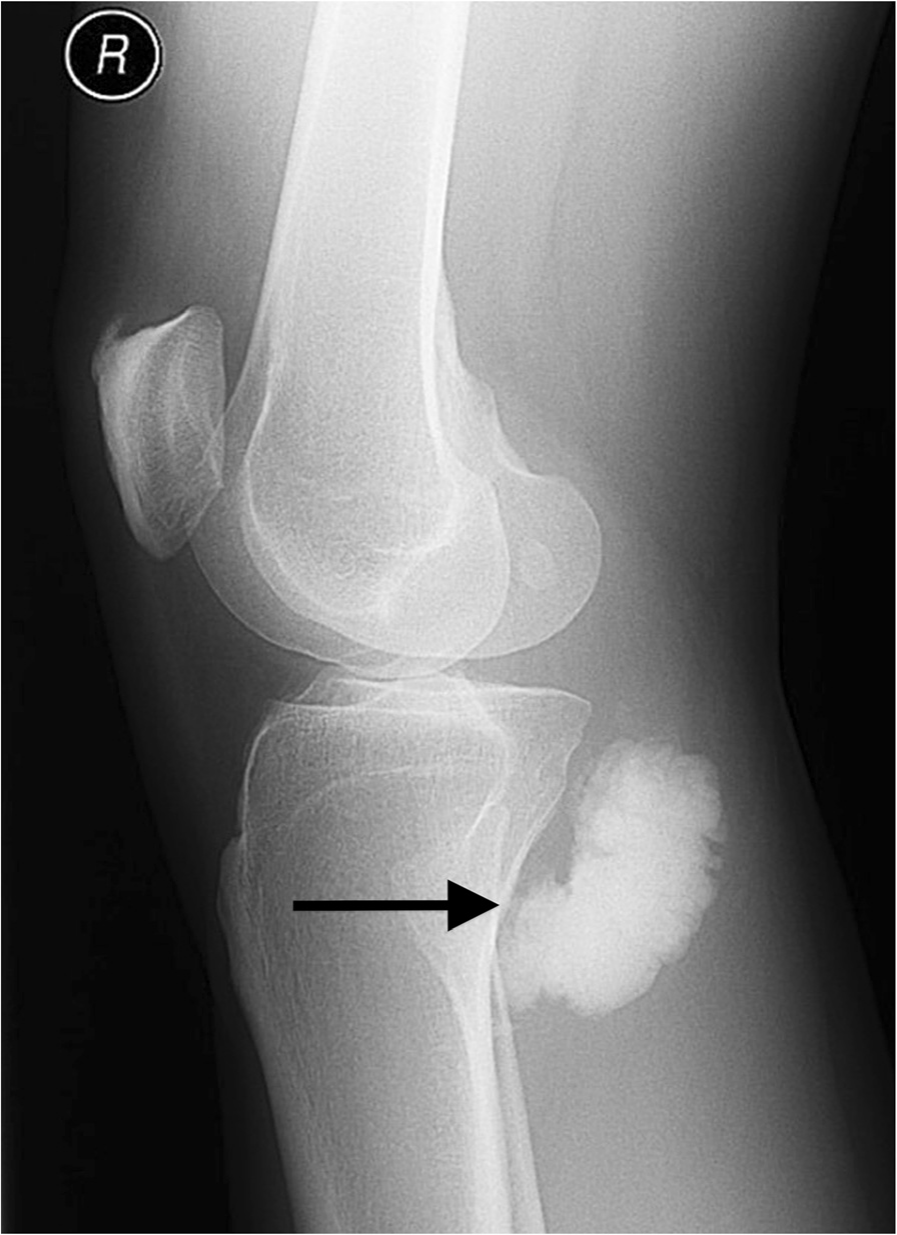
Lateral plain radiograph of the lesion.

Plain radiographs with anterior-posterior (Figure 1) and lateral (Figure 2) view of the right knee showed a lobulated and fully calcified mass, located dorso-medially to the tibial plateau. Radiopacity of the lesion was slightly higher compared to the adjacent bone of the metaphyseal tibia.

On magnetic resonance (MR) images a homogeneous and lobulated yet relatively well-circumscribed juxtacortical mass was visualized. The lesion was located close to the bone and between the medial head of the gastrocnemicus muscle, which was slightly displaced dorsally, and the pes anserine.

**Figure 3:**
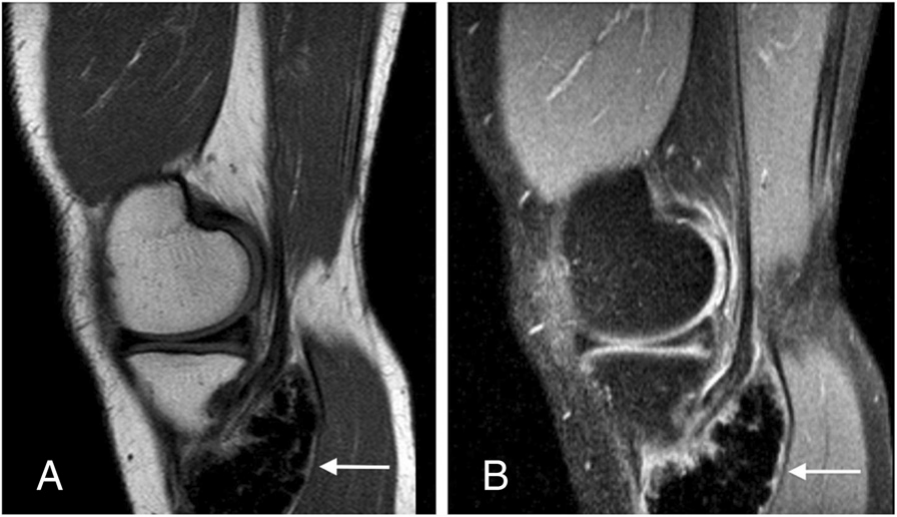
T1-weighted (A) and T2-weighted (B) sagittal MR image of the lesion (arrow).

T1-weighted sagittal images (Figure 3A) showed almost isointense signal compared to adjacent cortical bone and homogeneous decreased signal intensity compared to the bone marrow of the tibia and skeletal muscles. T2-weighted sagittal images demonstrated a slight increase in signal intensity of the proximally located tendon of the semimembranosus muscle, concordant to tendinitis (Figure 3B).

**Figure 4:**
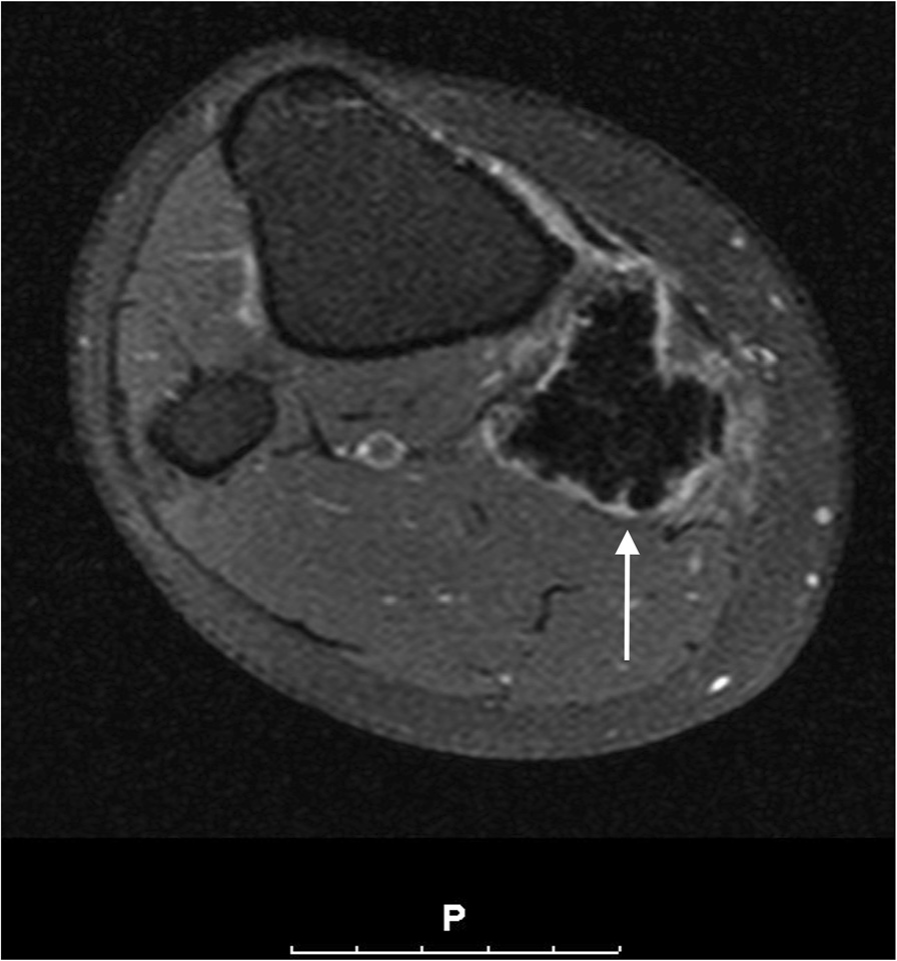
T1-weighted SPIR sequence, transversal cross-sectioning with gadolinium contrast medium enhancement.

**Figure 5:**
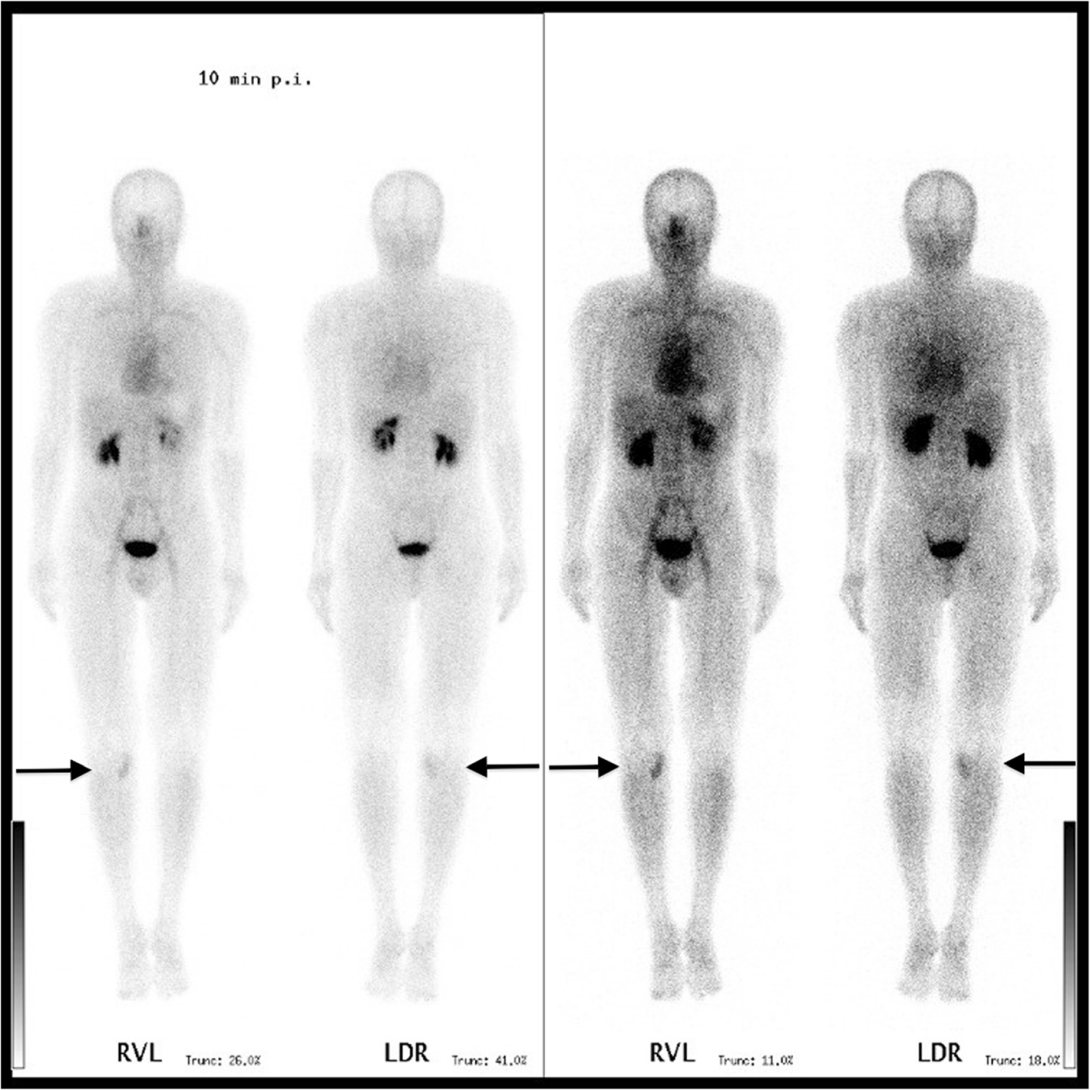
Whole-body bone scintigraphy.

The lesion showed only peripheral contrast enhancement after gadolinium contrast medium administration (Figure 4). On whole-body Tc-99 m bone scintigraphy, an increased uptake at the proximal, medial part of the right lower leg was seen (Figure 5). The lesion presented a distinct hyperemia during blood-pool and bone phase.

Radiographically, maligancy could not be ruled out. Surgical biopsy was indicated to obtain a histologically verified diagnosis. Surgery was accomplished in a prone position and excisional biopsy with marginal resection of the lesion was performed. Macroscopically, a thin synovium-like membrane covering the bone-like lesion was discovered and resected. A second, smaller nodule attached to the cranial pole of the primary lesion was also excised in the process. Both specimens were sent for histopathologic analysis.

**Figure 6:**
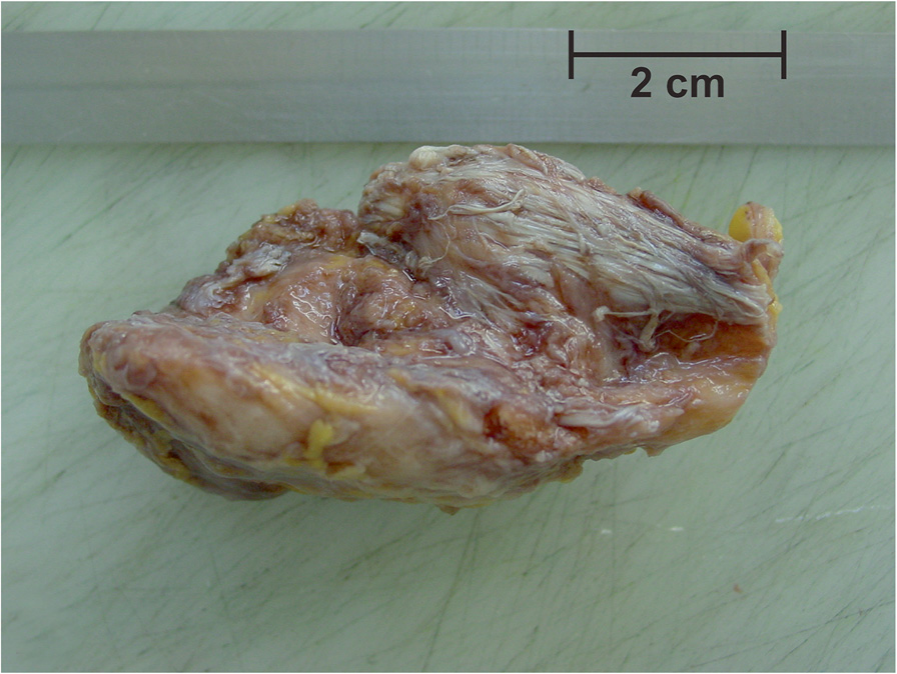
Macroscopic specimen after excisional biopsy.

**Figure 7:**
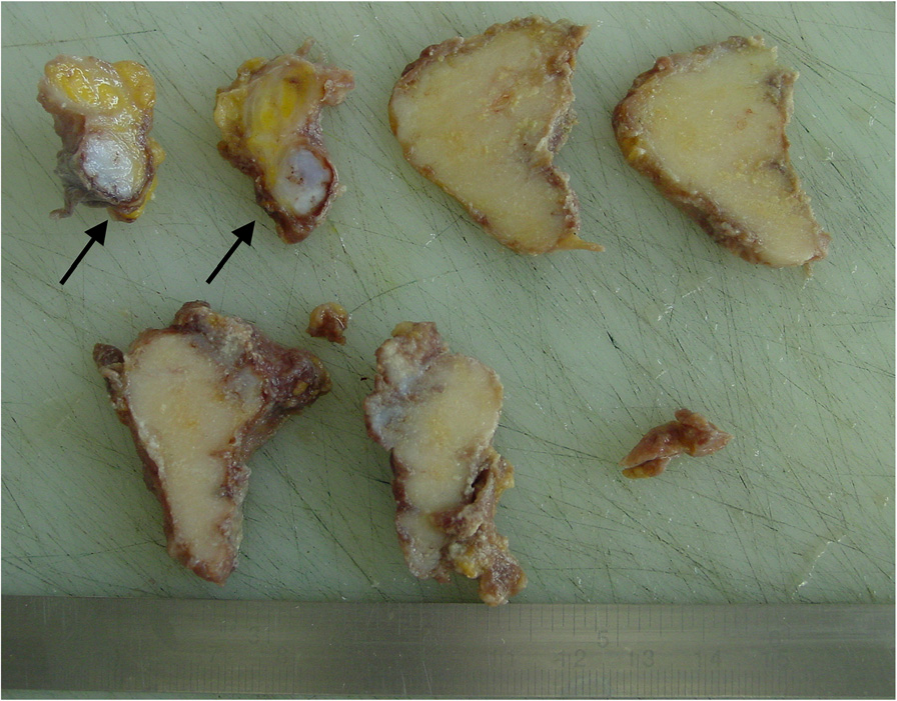
Sawcuts of the lesion and the attached tendon-like soft tissue nodule (arrows).

Histopathological examination of the first surgical specimen showed a spindle-shaped 7×3.5×3.5 cm large, ossified piece of tissue (Figure 6). A small piece of soft tissue consisting of muscle fibers and tendon-tissue was attached. On both, the cranial and caudal pole, small areas (max 0.6 cm) of chondromatous tissue were found. Sawcuts of the lesion revealed a homogenous, grey-white, and firm texture of the specimen with absence of necrosis or hemorrhage (Figure 7). Microscopically, compact osseous tissue adequate to newly-formed bone was found.

**Figure 8:**
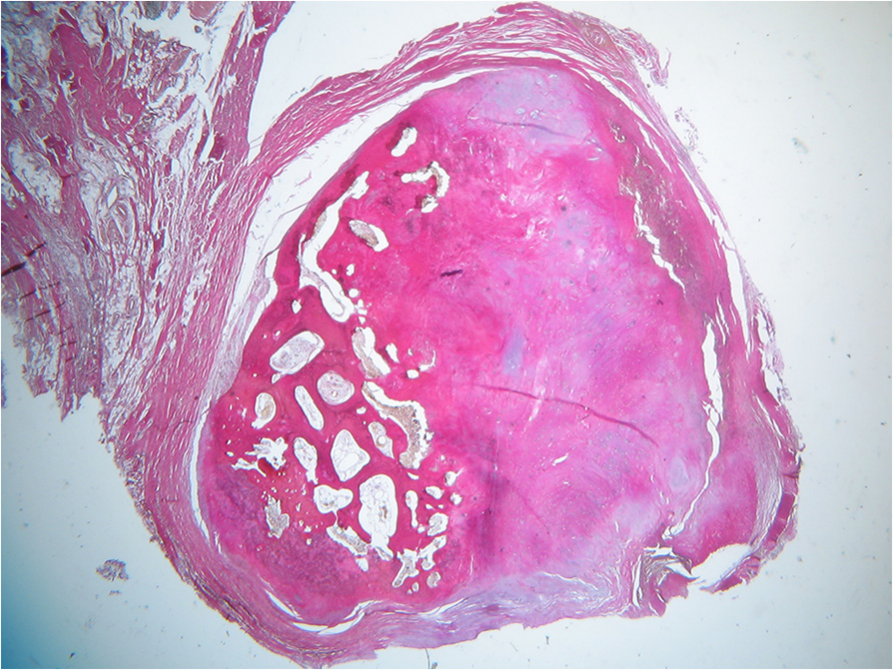
Second specimen, EVG-staining, 2.5× microscopic magnification.

The second specimen consisted of tendon-like tissue with irregular shape and appended fat-tissue. The size was 4×2×2 cm; the sawcut (Figure 7, arrows) showed grey-white disseminated calcifications. Microscopic examination revealed synovial tissue with chronic inflammatory changes and irregularly incorporated cartilaginous sections (Figure 8). Immuno-histochemical analyses were not conducted on either specimen.

**Figure 9:**
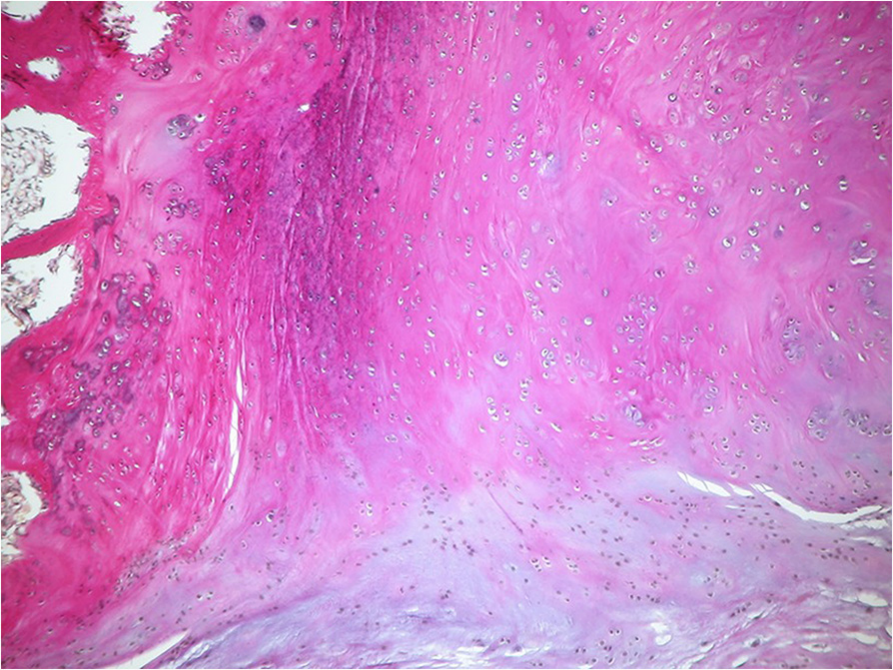
EVG-staining, 16× microscopic magnification of the hyalinous cap of the primary lesion.

The critical report noted an extensive formation of osseous tissue with tiny hyalinous caps for the primary lesion. These caps showed benign cartilage with cells arranged in a vertical pattern (Figure 9). For the second sample, a metaplasia of synovial tissue concordant to synovial chondromatosis was diagnosed. These findings did not support the differential diagnoses of calcifying tendinitis, tumoral calcinosis, or myositis ossificans. Parosteal osteosarcoma could not be ruled out completely by standard histopathological examination, though.

In this case, the patient remained free of symptoms and recurrence 2.5 years after surgery. Clinical examination and repeated imaging of the patient’s right knee is continuously performed every 6 months for a period of 5 years.

## Discussion

After clinical examination and imaging, a number of differential diagnoses for extra-osseous, heavily mineralized masses had to be taken into consideration. These included (giant synovial) osteochondroma, myositis ossificans, tumoral calcinosis, and parosteal osteosarcoma [3].

Giant synovial osteochondroma is a rare benign tumor that can be found mostly intra-articulary in the vicinity of the knee and hip joint. Besides trauma [4], an extra-synovial metaplasia of the outer layers of the capsule [5] is discussed as causes for this lesion. Yet in most cases, synovial chondromatosis is suspected to be the underlying reason. This arthropathy was first classified by Milgram in 1977 [6]. Edeiken complemented the original classification in 1994 by adding giant synovial osteochondroma as final stage of synovial chondromatosis [7].

The macroscopical and radiologic appearance of the lesion, either lobulated or smooth, depends on the potential genesis. A conglomerate of multiple small synovial chondromas is supposedly able to create one single coherent, lobulated cartilaginous giant osteochondroma. Otherwise, the continuous growth of a singular synovial chondroma is discussed to lead to a smooth and unruffled circumscribed tumor. In both scenarios, the cartilaginous structure of the synovial giant osteochondroma can be replaced completely by bone through enchondral ossification [6]. Nutritive supply of these progressively growing lesions is presumably coming from residual connections to the synovial membrane or by diffusion from the synovial fluid [7].

A review of the literature shows that studies to this entity are limited to case reports [4,5,7-12]. Besides an intra-articular form, an equally rare extra-synovial form does exist. It preferentially appears in the tendon sheath-rich areas of hand and foot and the knee. Thus, it is important to point out that a single, giant synovial osteochondroma, as a massive form of synovial chondromatosis, can potentially arise from any synovial tissue. Common synonyms used for giant synovial osteochondroma are extra-skeletal chondroma, ossifiying chondroma [5], intracapsular or para-articular chondroma [9], and extra-osseous osteochondroma [13]. Rarely, a malignant transformation from synovial chondromatosis into a synovial chondrosarcoma has been reported [14,15].

The differentiation between an extra-/para-articular or intra-articular giant synovial osteochondroma has to be established by the localization seen on preoperative imaging and encountered during surgery. It has to be noted that synovial covering of a chondromatous mass may derive from the tendon-sheaths or synovium of the popliteal bursae as well.

Myositis ossificans is a benign, self-limiting, solitary, ossifying soft-tissue disease typically occurring within the muscles of the upper and lower extremities [16]. It is commonly associated with soft tissue trauma, although in almost one-third of the cases there is no history of injury [17]; the pathogenesis of this lesion is unknown as well. The term heterotopic ossification is used as a synonym. Symptoms start with a painful swelling and local irritation with hyperthermia and diffuse edema [18]. The appearance of myositis ossificans on images varies, depending on the stage of development. Plain radiographs are initially negative. After 2-4 months the mass develops its typical feather-like feature, consisting of thin bony trabeculae. The heterotopic bone is usually more dense at the periphery of the mass, creating sharply defined borders. With ongoing maturation the mass becomes more ossified and resembles reparative bone callus. At the end, the lesion has the features of mature bone. Histologic proof of the diagnosis is given by the zoning phenomenon, where ossification and maturation progress from the periphery to the center [18]. This provides an important differentiation to (parosteal) osteosarcoma, where maturation of the lesion proceeds *vice versa* and presents a reverse zoning phenomenon. Surgical trauma might cause recurrence and marginal excision is only indicated after the lesion has matured.

Tumoral calcinosis is a very rare benign soft tissue calcification. Lesions consist of massive calcium deposits mostly occurring at the para-articular soft tissue [18]. The disease preferably affects the black population from birth until 20 years of age. In most cases local pain is the leading symptom [19]. Often the lesion is localized at multiple sites; large joints such as shoulder, hip, and knee joints are more often affected. Imaging shows a grape-like cluster of rounded calcified masses, separated by radiolucent septae [18]. As described for calcifying tendinitis, the histological differentiation is clear. Active foreign body reaction, including the presence of macrophages and giant cells, is bordered by granular calcified material. The etiology of this disease remains unclear [19,20]. Several metabolic diseases (in particular secondary hyperparathyroidism through chronic renal failure) accompanied by an imbalance of the calcium/phosphate homeostasis are being held responsible for the occurrence of this lesion [21]. Still, blood test may show normo-calcemic parameters [22]. Causal treatment of systemic disorders must come before surgery, though surgical excision is indicated with symptomatic lesions.

**Figure 10:**
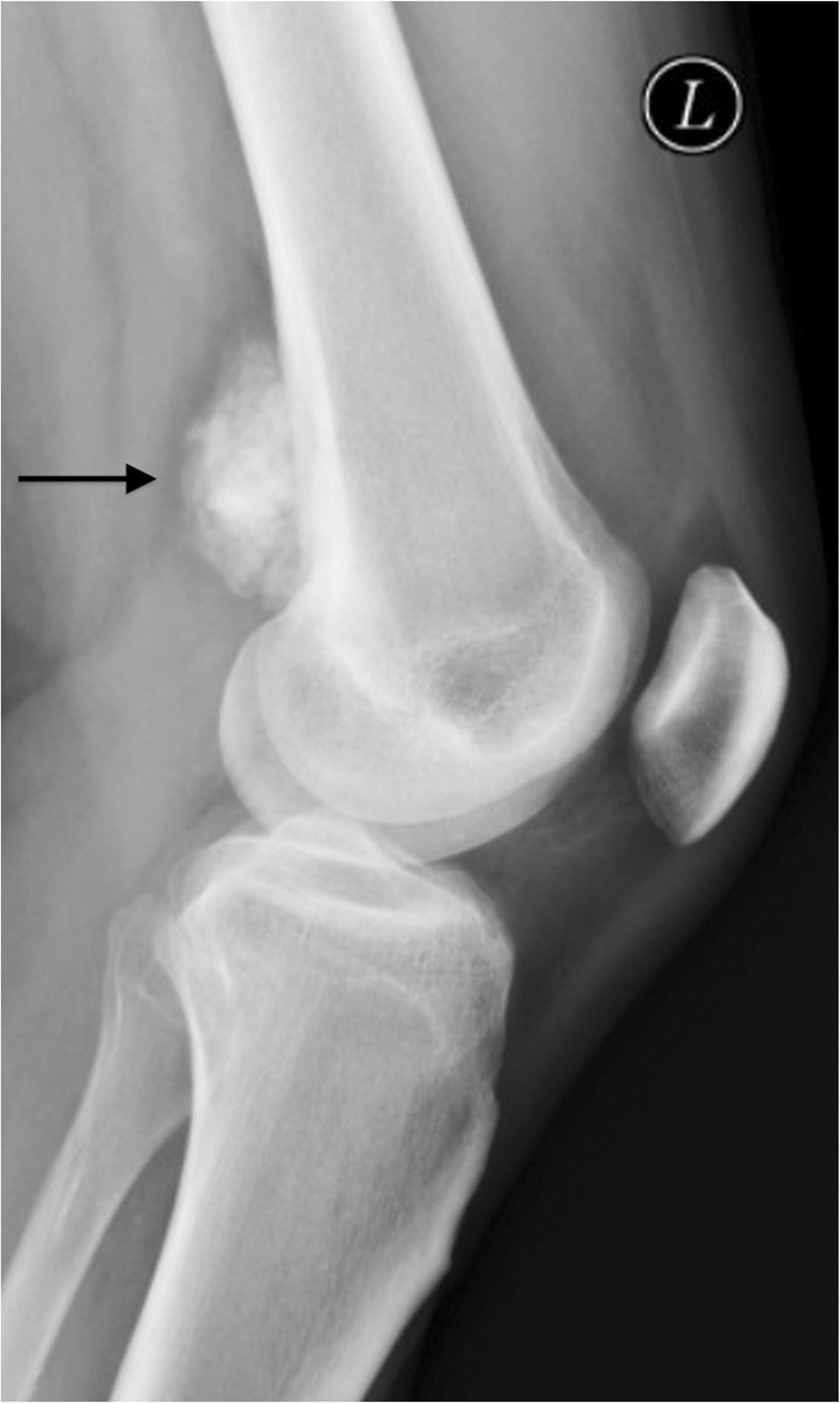
Exemplary lateral radiograph of a parosteal osteosarcoma, loco typico.

Parosteal osteosarcoma originates at the surface of the bone. Sites of predilection are the distal femur (planum popliteum), and less frequently, the proximal tibia and proximal humerus [18]. Parosteal osteosarcoma is a rare neoplasm, accounting for 4-5% of all osteosarcomas and affects a slightly older patient population compared to classic osteosarcoma. Age distribution is between 20 and 40 years of age. Depending on localization, symptoms are mostly non-specific and might include a mechanical limitation in flexion of the knee if it is located ‘loco typico’ at the posterior aspect of distal femoral or proximal tibial metaphysis. The growth of the lesion is slow and the symptoms’ duration frequently exceeds several years. Imaging reveals a radiodense, lobulated mass with radiopacity diminishing peripherally, as an exemplary radiograph of a parosteal osteosarcoma with origin from the distal femoral metaphysis clearly shows (Figure 10). There usually is wide contact to the underlying cortex. Histopathology shows immature bone in different stages of maturation to lamellar bone and an abundance of spindle cells. This fibrous stroma is interspersed with osseous trabeculae and collagen fibers and shows low mitotic activity. Focally, tumor cartilage might exist [18,23]. A cellular cartilage cap external to the osseous portion of the lesion can be found in about 25-30% of cases. This occurrence can result in the pathologic misdiagnosis of osteosarcoma as osteochondroma [24]. Determining malignancy of a parosteal osteosarcoma may present some difficulty as both the clinical course, imaging, and standard histological analysis do not necessarily lead to a specific diagnosis.

This is why parosteal osteosarcoma remains hard to differentiate from benign entities [25-27]. However, new findings in immunohistochemistry with determination of MDM2 and CDK4 markers show promising results in distinguishing parosteal osteosarcoma from benign clinical and histological mimics [28].

## Conclusion

Assessing dignity of extra-osseous, mineralized masses by imaging alone can provide some difficulty. Benignant lesions in the vicinity of the knee can show remarkable similarities to malignancies and *vice versa*. For a histological clarification of uncertain lesions, a biopsy is indicated, even if the symptoms are minor. In differential diagnostics between osteochondroma and parosteal osteosarcoma, histological analysis is not yet routinely able to provide diagnostic certainty in all cases. Therefore, surgical therapy with complete excision and narrow follow-ups are strongly recommended until further developments in immuno-histochemical analyses give absolute certainty.

## Consent

Written informed consent was obtained from the patient for publication of this case report and any accompanying images. A copy of the written consent is available for review by the Editor-in-Chief of this journal.

## Competing interests

The authors declare that they have no competing interests. No benefits in any form have been received or will be received from a commercial party related directly or indirectly to the subject of this article.

## Authors’ contributions

AT carried out the surgery, performed the study, and prepared and revised the manuscript. UL and FP helped to draft the manuscript, examined the patient for various check-ups, and helped with an up-to-date literature review. FL helped to draft the document and handled the illustrations and picture-work. RE revised the manuscript critically for important intellectual content and helped to draft the manuscript. HM was crucial in the revision process of the reviewed manuscript and helped to check the quality of written English. All authors read and approved the final manuscript and its revision.
